# A psychometric study of an executive function assessment instrument (TDI-FE)

**DOI:** 10.1186/s40359-023-01373-2

**Published:** 2023-10-14

**Authors:** Sérgio Kakuta Kato, Flávia Amaral Machado, Machline Paim Paganella, Leia Gonçalves Gurgel, Vanessa Kaiser, Gabriela Bertoletti Diaz, Adriana Jung Serafini, Nelson Hauck Filho, Caroline Tozzi Reppold

**Affiliations:** 1 Psychological Assessment Laboratory, Community Health Department, Graduate Program in Rehabilitation Sciences), Federal University of Health Sciences of Porto Alegre (UFCSPA), Porto Alegre, Brazil; 2 PUCRS. School of Technology, Pontifical Catholic University of Rio Grande do Sul, Porto Alegre, Brazil; 3 Life Sciences Knowledge Area, Institute for Health Researches, University of Caxias do Sul (UCS), Caxias do Sul, Brazil; 4 Psychological Assessment Laboratory, Federal University of Health Sciences of Porto Alegre (UFCSPA), Porto Alegre, Brazil; 5 Psychological Assessment Laboratory, Department of Psychology, Federal University of Health Sciences of Porto Alegre (UFCSPA), Porto Alegre, Brazil; 6 Graduate School of Psychology, Universidade São Francisco, Campinas, Brazil

**Keywords:** Executive functions, Validation studies, Child development, Psychometrics

## Abstract

**Background:**

This study aims to present and discuss the psychometric properties of executive functions, which were measured using the TDI-FE instrument. The analysis encompasses its internal structure, potential sensitivity to fatigue factors, relationships with external criteria, and diagnostic accuracy.

**Methods:**

The study sample comprised 382 students from Brazil, aged 6–8 years. Child development variables were screened using the TDI-FE and gold standard tests (Cancellation Attention and Trail Making Tests). The proposed scale comprised four activities: a test with fruit images with three tasks, and one memory game.

**Results:**

The one-factor model of EF of the TDI-FE failed to fit to the data. However, fit substantially improved once a latent fatigue factor was controlled in the model. The latent factor of EF assessed by the TDI-FE tasks was coherently associated with a series of external variables, including two popular collateral measures of EF. The diagnostic accuracy was reasonable, and a cut-off of 37 points produced 70% of sensitivity and 60% of specificity.

**Conclusion:**

Results indicated that the TDI-FE demonstrated sound psychometric properties and diagnostic accuracy, then consisting of an efficient alternative for the assessment of EFs in early childhood education. The study also proved the need to control for response biases such as fatigue in the latent variable models of EF. The TDI-FE is notable because of its low cost and easy application, and it might fulfill a need for instruments for individuals from different contexts at this stage of development in Brazil.

## Background

The brain as an organization that performs cognitive activities is one of the main focuses of research in Neuropsychology. The execution of a task and the neurocognitive processes involved in it are crucial for identifying essential functions linked to human social behavior: the different executive functions [1–3].

Executive Functions (EFs) enable individuals to perform actions that offer greater autonomy, and the power to control a range of interactive cognitive processes concerning the frontal lobe that are essential for engaging in complex, novel, and goal-directed behaviors [4]. The concept of executive functions indicates a set of cognitive abilities that control and regulate other more basic capacities (such as attention, memory, and motor skills) and plan to guide goal-directed behavior or problem-solving [1, 3–5].

In her study, Diamond presented a theoretical model that is widely used in the existing neuroscience literature [6]. The model determines three major components of EFs: inhibition, working memory, and cognitive flexibility. Inhibition involves inhibitory control, including self-control (behavioral inhibition) and interference control (selective attention and cognitive inhibition). This construct comprehends the control of attention, behavior, thoughts, and emotions to modulate a strong internal predisposition or external attraction to a certain action.

Impulsivity and its motor aspects are related to inhibitory control and are characterized by cognitive and behavioral manifestations that are usually measured by “perseverative errors” or by errors in responses to non-target stimuli that indicate a lack of inhibitory control. Contrarily, non-planning impulsivity is directly related to cognitive inhibition (the ability to inhibit automatic thoughts or responses voluntarily) and decision-making (a process that requires choosing one of several alternatives) [7]. It is more common for impulsivity to occur in childhood when the cognitive maturation process is still developing [8, 9].

The second component, working memory can be categorized into verbal (e.g., auditory) and non-verbal (e.g., visuospatial) memories; it helps retain learned information and process this data to make associations between contents, comprehension, problem solving, and so on. This is given by the maintenance, temporary manipulation, and updating of information arising from the setting [10]. In this process, there is a transitional storage of the information that is relevant to a particular task which maintains the goal of an activity and actively makes the information relevant, and then to subsidize other cognitive functions [4, 7].

The third component, cognitive flexibility, is defined as the ability to search for a solution in a challenging situation by changing the perspective or focus and flexibly adjusting to new demands, rules, or goals [6, 11]. It points to an individual's capacity to adapt their choices to circumstances, which demands change or alteration in their course of actions or thoughts based on the demands of the environment.

The relationship between EFs and academic performance has been widely documented in existing literature, and there is robust evidence indicating the influence of EFs on social-emotional and cognitive development at various stages of life [8, 12, 13]. A study investigating models demonstrated the predictive power of EF on reading and arithmetic performance two years later [14]. Another study investigated the development and stability of EFs during the transition to first grade and its relationship with academic performance, highlighting that the transition period to first grade can be an important period to promote the development of EF, which in turn can support the prevention of later school problems [15]. Furthermore, another study provided data on a specific contribution, during childhood, of an inhibitory type to fluid intelligence and contributed with evidence that EFs are considered a predictor of performance in various assessed school domains, both in the advancement of content and in the increase of complexity in tasks. Especially in aspects related to language, such as written production, with the improvement of academic skills performance in children and adolescents, when re-evaluated after specific intervention [8]. As an example, there is a school activity that requires autonomy and more specific attention and organization resources, requires determination of objectives and planning to update them, in addition to flexibility to seek alternative strategies, requiring adequate functioning of the EF [8, 12]. Thereby, EFs are considered fundamental in the regulation of social, intellectual, and emotional skills; therefore, any alterations or deficits in any of the components of EFs can cause delays in school readiness and difficulties in the learning process [13, 16–18].

These difficulties in the learning process related to EFs are associated with any of the domains related to the process of acquiring new skills, such as listening comprehension, language expression, basic reading skills, reading comprehension, written expression, mathematical calculation, or mathematical reasoning [19]. Among the components of EFs, working memory is noteworthy as it is an important predictor of all academic domains, especially mathematical skills [20]. This reinforces the importance of conducting early assessments of EFs aimed toward children in the initial stages of childhood education, mainly because EFs are essential in the process of acquiring new skills.

In this sense, the evaluation of EFs in children is advantageous, as it can help identify difficulties in their development and collaborate in the prevention of school failure and obstacles in the acquisition of skills. In Brazil, the offer of free instruments is limited and not available in the public health and education systems. The Child Development Surveillance Manual, published in the Child Health Booklet, is commonly used as a measure for assessing child development, through neuropsychomotor development milestones, directed only to early childhood. However, this record has low accuracy and lacks updates by healthcare professionals in aspects related to motor and cognitive development [21]. In the area of education, there is no instrument accessible to teachers and pedagogical coordinators in the public school system. In this situation, educators use qualitative assessments of reading and writing skills to identify learning difficulties, however other areas, such as EFs, may be interfering in this process [22]. Based on this perspective and the setting in Brazil, the use of an inexpensive and easily applicable screening instrument becomes a viable alternative for the early detection of problems in children’s development [23, 24].

Another noteworthy concern pertains to the issue of fatigue experienced by respondents during the administration of lengthier tests. This phenomenon has been observed in various contexts, where individual variations may interplay with the test's duration, leading to subjective fatigue [25] and potentially compromising the quality of responses provided to the test items [26]. Of particular significance is the impact of fatigue within the realm of assessing executive functions. This factor holds the potential to significantly distort the performance outcomes, especially among younger individuals [27]. For instance, a child subjected to two tests evaluating identical neuropsychological constructs but differing in the number of items or tasks could achieve disparate results due to the influence of fatigue. Considering that conventional assessments of executive functions tend to be lengthier in nature, it becomes imperative to incorporate a statistical mechanism to control for the presence of fatigue. By doing so, the variance introduced by fatigue can be mitigated, preventing its contamination of the data, and safeguarding the integrity of the findings derived from subsequent data analyses.

The objective of this study was to analyze and discuss the psychometric properties of Child Development Screening - Executive Functions (TDI-FE), including its internal structure, potential contamination by a fatigue factor, relationships with external criteria, and diagnostic accuracy. We hypothesized that, despite capturing a general executive functions factor, the later tasks of the TDI-FE might have performance also influenced by a fatigue factor.

## Methods

This study is part of an investigation to construct, validate, and standardize an instrument called *Bateria Multidisciplinar de Triagem do Desenvolvimento Infantil* (TDI) (In Portuguese: Multidisciplinary Screening Battery for Child Development). In addition to EFs, this set of tests covers the “language” and “motor skills” dimensions. Information regarding development and validity of the scale instrument was described elsewhere [28].

### Participants

The study sample consisted of 382 students from Brazil in the first, second, and third years of primary education, which is early childhood education [29, 30] in public state schools in the city of Porto Alegre. The sample size was determined by calculating the total number of children enrolled in public schools in Porto Alegre, according to the data from the Rio Grande do Sul State Department of Education. Moreover, we made efforts to ensure that the sample size would not fall below the popular rule of thumb of collecting responses 10 times the number of items to be assessed in the instrument. Therefore, the required sample size was 340 children (14 items in tasks F1 to F3 and 20 trials in task F4). With the authorization of the Department of Education, the schools were selected from all regions of the city to increase the representativeness of the sample.

The selection criteria in this study were: children between the ages of 6 years, and 8 years and 11 months, who were regularly enrolled in a public school in Porto Alegre and had both an informed consent form signed by a parent or legal guardian and signed an assent form for minors. Children with neurological impairment (according to the medical records provided by the school), or those who had not completed the test battery were excluded.

### Instruments

#### Instrument used to estimate accuracy

**Fig. 1:**
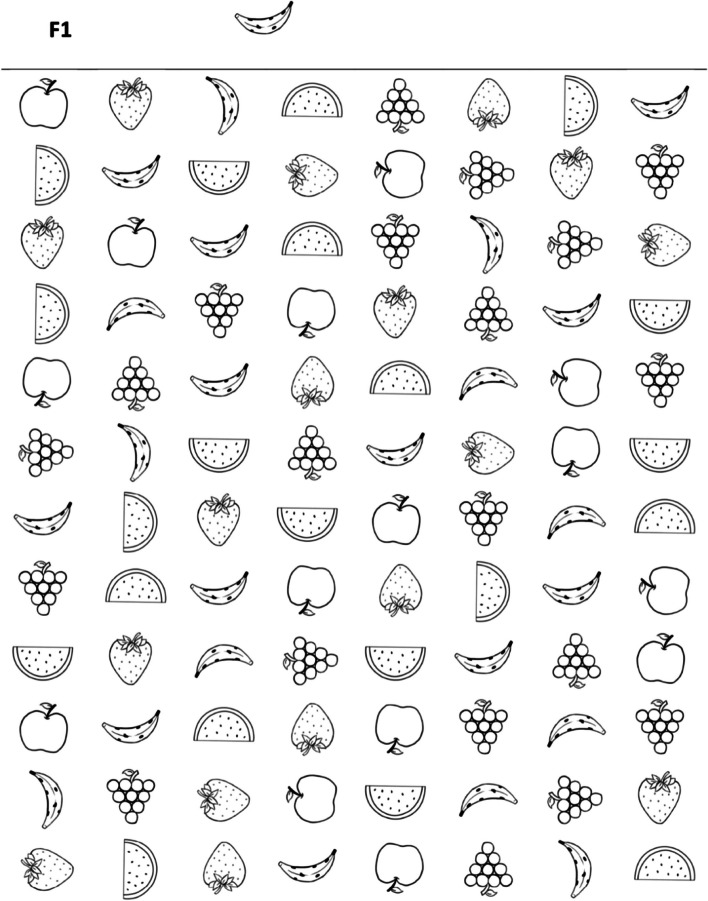
The Fruit Test - Task F1

**Fig. 2:**
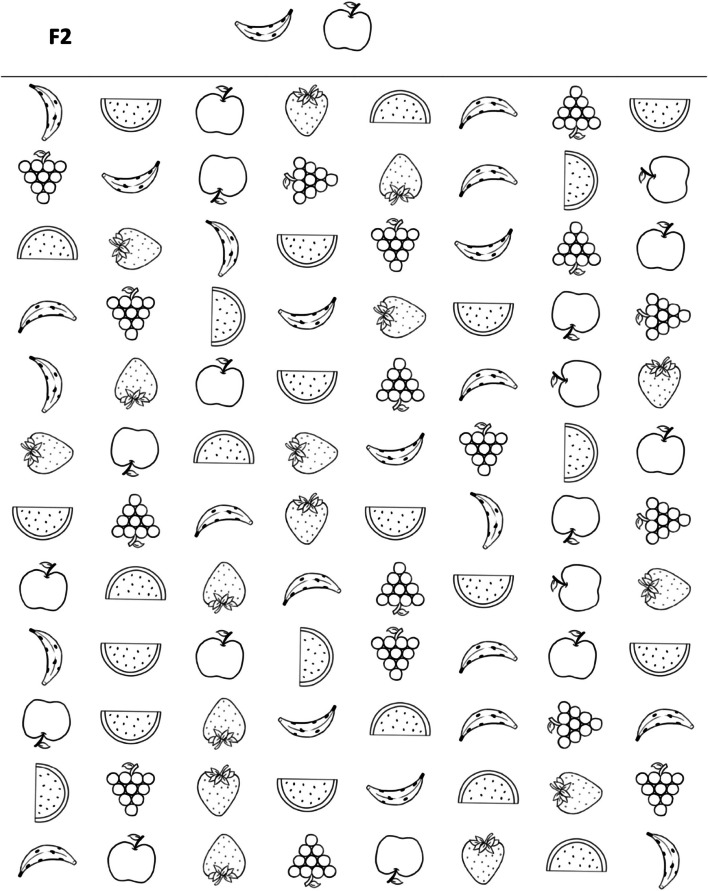
The Fruit Test - Task F2

**Fig. 3:**
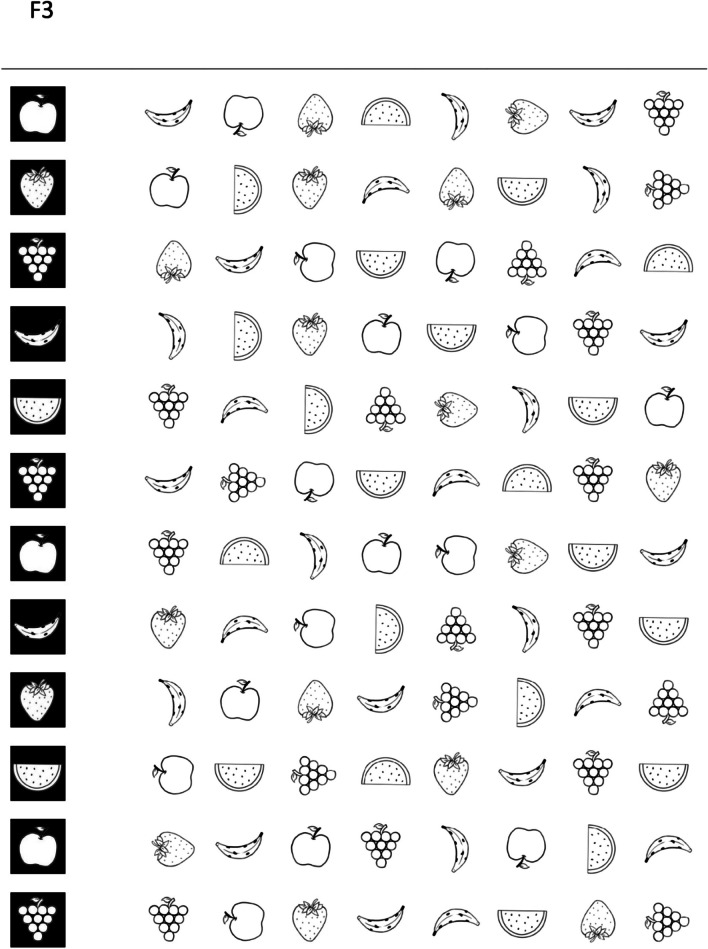
The Fruit Test - Task F3

**Fig. 4:**
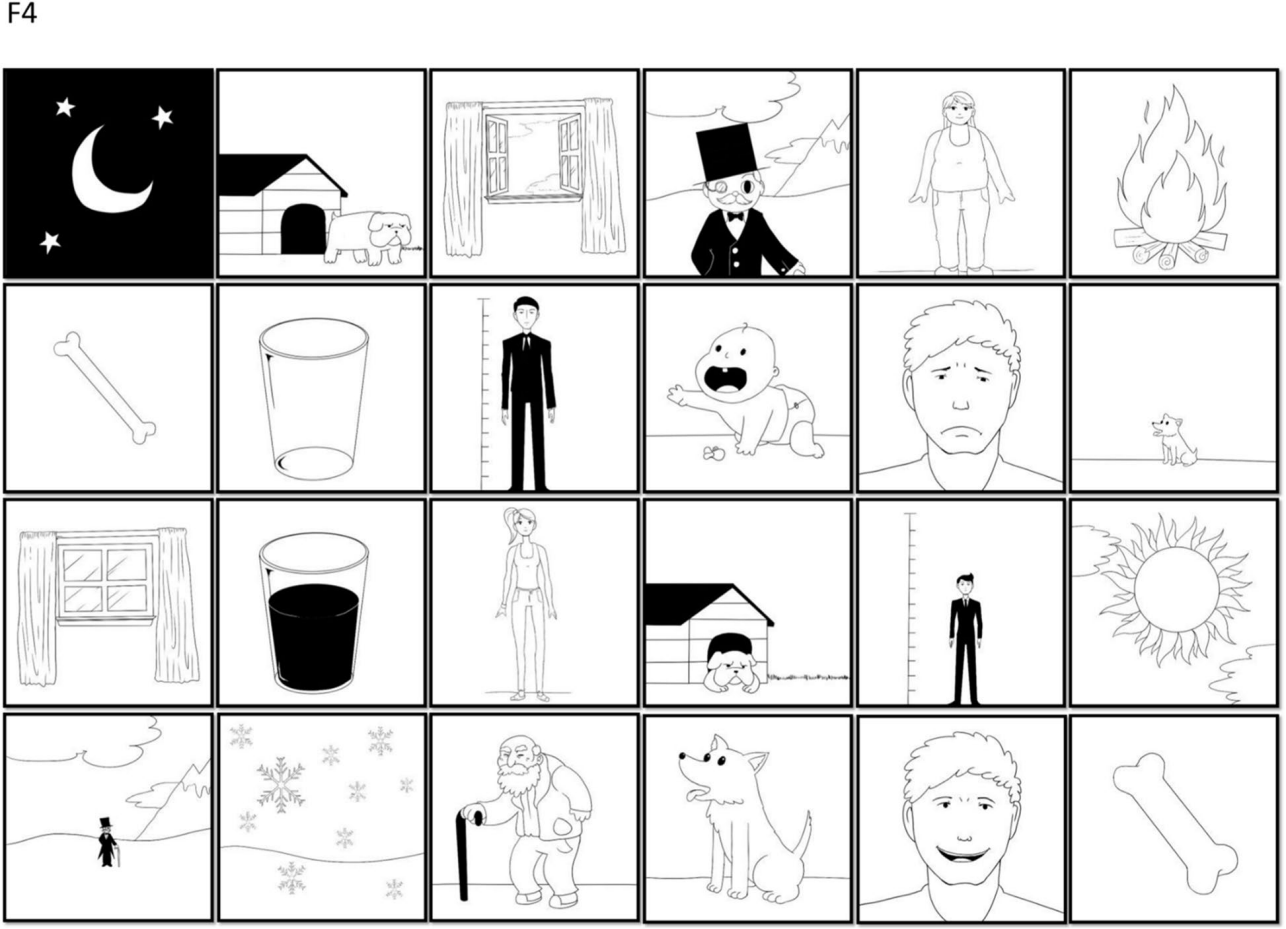
The Memory Game - Task F4

The TDI-FE is part of a multidisciplinary screening battery that assesses EFs, motor skills, and language in children in early childhood education [28]. Specifically, this instrument considers three constructs and is composed of four activities: F1, F2, F3, F4 (Figs. 1, 2, 3, 4). The Fruit Test consisted of inhibitory control tasks which involve cognitive inhibition and selective attention. It included three printed matrices (F1, F2, F3) with distinct types of stimuli. Each matrix presented a level of difficulty and contained 96 images of fruits distributed throughout 8 columns and 12 rows. In the first matrix (F1), the child was instructed to find and mark as many images as possible according to the targeted image. In the second matrix (F2), the child was instructed to find and mark a pair of targeted images. The total number of correct markings in both matrices was considered for arriving at a conclusion, and the time limit was 40 seconds. In the third matrix (F3), the child was instructed to find and mark the targeted image at the beginning of each row. The correct number of markings per row (from 1 to 12) was considered and the time limit was 40 seconds. The Memory Game (F4) assesses cognitive flexibility and working memory. It is a memory game of opposites that measures memory capacity for a specific task while opposite drawings are associated with the task. The game was set up based on a predetermined template, and the participant was instructed to look at the position of the cards for one minute, after which, the drawings were hidden; the participant had 20 attempts to find the 12 pairs of opposite figures. The numbers of correct pairs and incorrect pairs found by the participant were considered.

### Instruments used to identify validity evidence based on convergent external variables

To identify validity evidence based on relationships with convergent external variables, along with TDI-FE, three other equivalent instruments that have been adapted and validated for the Brazilian population were also applied.

#### The *Cancellation Attention Test (CAT)* (in Portuguese: *Teste de Atenção por Cancelamento*) [31]

In the Cancellation Attention Test, the participant had to mark all stimuli that were equal to a previously determined target stimulus. It was composed of three printed matrices with diverse types of stimuli. The CAT is a figure cancellation test with a printed matrix containing six distinct types of stimuli in black (geometric figures) set on a white background, distributed across 18 rows, with each row consisting of 20 figures. The participant marked the target stimulus whenever it appeared within a maximum time limit of one minute, which assessed selective attention. The validity evidence for CAT was obtained for children aged between 4 and 14 years.

#### The *Trail Making Test (TMT) - Parts A and B* (in Portuguese: *Teste de Trilhas A e B*) [32]

This Test assessed mental flexibility, visual search, and motor function. It was divided into two parts. Part A comprised two sheets (one for letters and the other for numbers): the first sheet presented 12 randomly arranged letters from “A” to “M” (absence of the letter “K”). The participants were instructed to connect the letters in alphabetical order. The numbers “1” through “12” were arranged randomly on the second sheet, and the child was instructed to connect them in ascending order. In Part B, letters (12 items) and numbers (12 items) were randomly arranged, and the child was instructed to connect them in an alternative manner (first letter, and then the number). The time limit was one minute for all the sheets. The test measured the correct answers, errors, and elapsed time from the scores of the TMT - Parts A and B and the results were according to the normative table.

#### The *Trail Making Test for Preschoolers (TMTP)* (in Portuguese: *Teste de Trilhas para Pré-escolares*) [33]

This test maintains the same objective as the original version, but it does not require knowledge of letters and numbers. It consisted of two parts. Only one type of stimulus was presented in the first part. There were two types of stimuli in the second part that had to be marked by the participants in an alternative order, similar to TMT. In part A of the test, the child was given a sheet with five images of dogs that had to be arranged in ascending size order, from “baby” to “daddy.” Part B depicted images of bones in the respective sizes of the dogs, and the child was instructed to match the dogs to their bones in the ascending size order by arranging them alternately. The child’s performance in each part was measured by sequence (number of items correctly connected in sequence) and execution time. Trevisan and Seabra obtained evidence of its validity and data standardization for children.

### Procedures

First, the study was presented to the Department of Education to authorize the research team’s access to the schools. In each selected school the principal investigator introduced the researchers, an overview of the procedures and method of data collection. Informed Consent Forms (ICF) were issued to the parents/guardians of the students through the students’ planners. A total of 660 forms were issued, and only 396 were signed (about 60%) by parents/guardians. Out of 396 children, 382 completed the battery of tests.

### Data collection

The data collection period spanned from October 2016 to April 2018. Two separate meetings were held with each child: one to administer the TDI-FE and the other to administer the TMT and CAT tests. These activities occurred during regular class periods based on each student’s availability. The average duration of each application was 20 minutes, and the applications were conducted by eight evaluators who had prior training in the application of the instruments. The school provided a space with adequate lighting and materials to administer the tests. The students responded to the questions on the forms given in each test. The response protocols were corrected and reviewed by two independent evaluators, and all protocols were corrected and typed by a third evaluator.

### Data analysis

First, Confirmatory Factor Analysis (CFA) was performed to analyze the internal structure of the TDI-FE using the weighted least squares means and variance adjusted (WLSMV). Subsequently, a second CFA was conducted to control for an intervening latent factor of fatigue. The second model diverges from the first model in a distinct manner. In this instance, an extra component has been integrated to account for the onset of fatigue experienced by students during the execution of these final tasks. This was accomplished by introducing a method factor exclusively linked to the concluding nine tasks of the TDI-FE. Notably, this method factor retained orthogonality, signifying its lack of correlation with the authentic executive functions factor. Method factors serve to encapsulate systematic variation that remains disconnected from the constructs under examination [34]. However, this variance possesses the potential to contaminate the data, subsequently confounding the outcomes derived from analytical investigations. Finally, the external factors age, the CAT, and the TMT - Parts A and B were incorporated into a Structural Equation Model as external variables. In these analyses, the following indices were considered: comparative fit index (CFI; must be ≥ 0.95), Tucker–Lewis Index (TLI; must be ≥ 0.95), and root mean square error of approximation (RMSEA; must be ≤ 0.06) [35]. In the final model, the estimates for the difficulty parameter of the items in task F3 were evaluated by the item response theory (IRT) graded response model [36]. Furthermore, to estimate diagnostic accuracy, we relied on ROC curve analysis, which calculates specificity and sensitivity and helps determine the optimal cut-off point for the proposed EF score. The grouping variable was children with and without deficits in EF according to the CAT and the TMT, as described later. The analyses were conducted using Mplus 7.11 and SPSS 22.0.

## Results

The study sample included 382 children aged between six to eight years who were enrolled in the 1^st^, 2^nd^, and 3^rd^ year of Primary Education. Table 1 presents the distribution of the variables in the sample.

**Table 1:** Distribution of the characteristic variables of the sample

	Frequency	Percentage
Sex		
Female	229	59.9
Male	153	40.1
Age		
6 years	114	29.8
7 years	134	35.1
8 years	134	35.1
Education		
1^st^ year	169	44.3
2^nd^ year	134	35.0
3^rd^ year	79	20.7

**Fig. 5:**
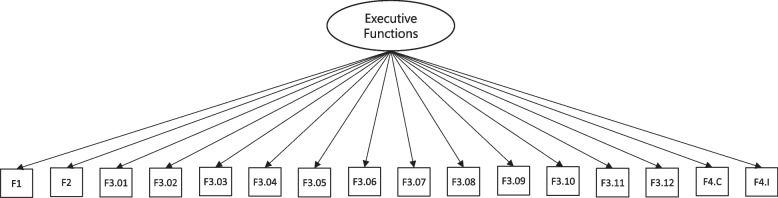
CFA diagram to evaluate the internal structure of the TDI-FE scale

The proposed scale is composed of four different activities: three tasks at varied levels of difficulty (F1, F2, F3) in the fruit test and the memory game (F4). These activities were consistently executed by the children in this order. The CFA was first conducted to test the one-dimensional model of a general factor of EFs, according to Fig. 5. The one-dimensional model did not achieve a good fit: χ^2^(209) = 2662.65, RMSEA = 0.175, CFI = 0.371, TLI = 0.402. In this analysis, a very strong residual correlation was observed between the last items of task F3, in which χ^2^ of the modification indices suggested the need to control for this effect in the model. These residual correlations are consistent with the fact that a potential fatigue factor would also account for the performance in the last tasks of the test battery. These last items could have their responses partially contaminated by a lack of motivation and attention among some students.

**Fig. 6:**
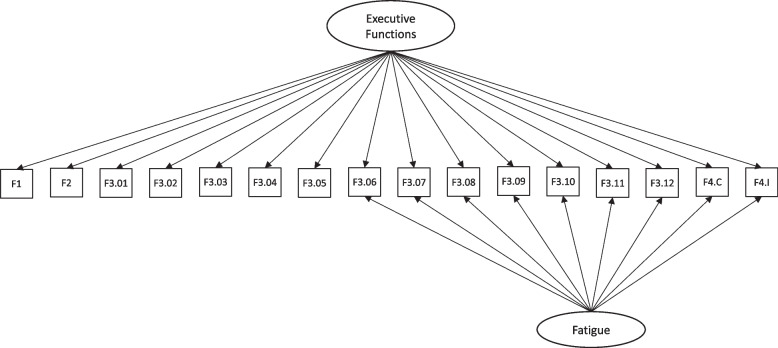
CFA diagram to evaluate the internal structure of the TDI-FE scale by controlling for the effect of the Fatigue factor

To control for this effect, a latent factor of fatigue was added to the model, as shown in Fig. 6. As hypothesized, the confounding fatigue factor exerted influence from indicator 6 of task F3, until item 12. Interestingly, a compensatory effect was found in which variance was increasingly explained by fatigue, and decreasingly explained by the EF factor. Model fit greatly improved, χ^2^(95) = 555.78, RMSEA = 0.113 CFI = 0.944, TLI = 0.929.

**Fig. 7:**
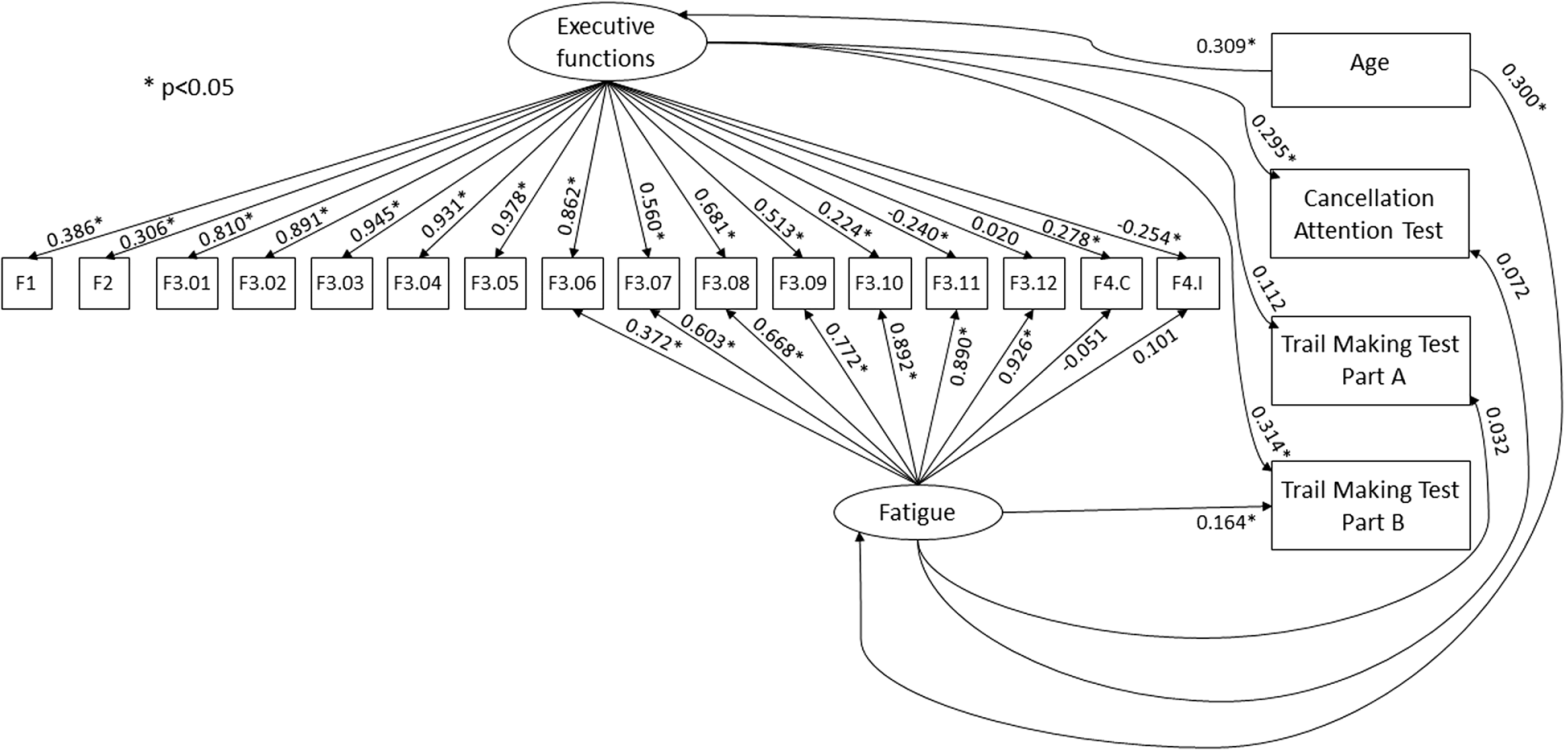
Structural Equation Model to assess Executive Functions in schoolchildren (age 6 – 8 years), predicting the Cancellation Attention Test and the Trail Making Test – Parts A and B

Next, we tested the connections between the two latent factors – EF and fatigue –, with the external variables age, Cancellation Attention Test, Trail Making Test Part A, and Trail Making Test Part B. Results can be found in Fig. 7, where error terms are omitted for the sake of simplicity. This final model achieved a reasonable fit: χ^2^(154) = 583.52, RMSEA = 0.084, CFI = 0.943, TLI = 0.929. Modification indices suggested that other tasks would be also slightly impacted by the fatigue factor. However, even if model fit would increase by adding such effects into the model, their size would be small and of little practical relevance, the reason why we decided not to include them in the model. In addition to the same factor loadings pattern observed in the model with only the internal structure, now other linear effects confirmed the interpretation of the EF and fatigue factors. The EF factor positively impacted scores on the Cancellation Attention Test, Trail Making Test Part A (non-significant), and Trail Making Test Part B. This indicates that the same EF that accounts for the performance in the TDI-FE also explains the performance in these other parallel tests. On the other way out, age positively predicted EF, indicating that older children display improved EF. The fatigue factor only predicted scores on the Trail Making Test Part B, with a very small effect size. However, it was positively predicted by age, indicating that older children were more prone to lose attention and motivation when taking the TDI-FE tasks.

Supplementarily, we conducted a separate graded response (IRT) analysis of the indicators that comprise the F3 task. This would aid to confirm if the stimuli were correctly assembled in a continuum of increased complexity. Results can be found in Table 2. The number of correct responses varies across indicators, the reason why they have a different number of difficulty parameters (b1, b2, and b3). As expected, the first item was the easiest. More specifically, the necessary theta level to have at least a 50% probability of one or more correct responses (b1) was a little more than one standard deviation (1.110) below the mean. By contrast, to have a 50% probability of giving one or more correct responses to item 12 required a much larger theta of 1.193. In sum, the results confirm the desired order in the complexity of stimuli that comprise the F3 task.

**Table 2:** Estimates for the difficulty parameter of the items in task F3

F3 Task Item	b1	b2	b3
1	-1.110	-	-
2	-0.943	-0.567	-
3	-0.654	-	-
4	-0.773	-0.397	-
5	-0.642	-0.400	-
6	-0.497	-0.234	-
7	-0.527	-0.359	-
8	-0.013	0.188	-
9	0.294	-	-
10	0.514	0.677	0.792
11	0.522	0.711	-
12	0.962	1.193	-

**Fig. 8:**
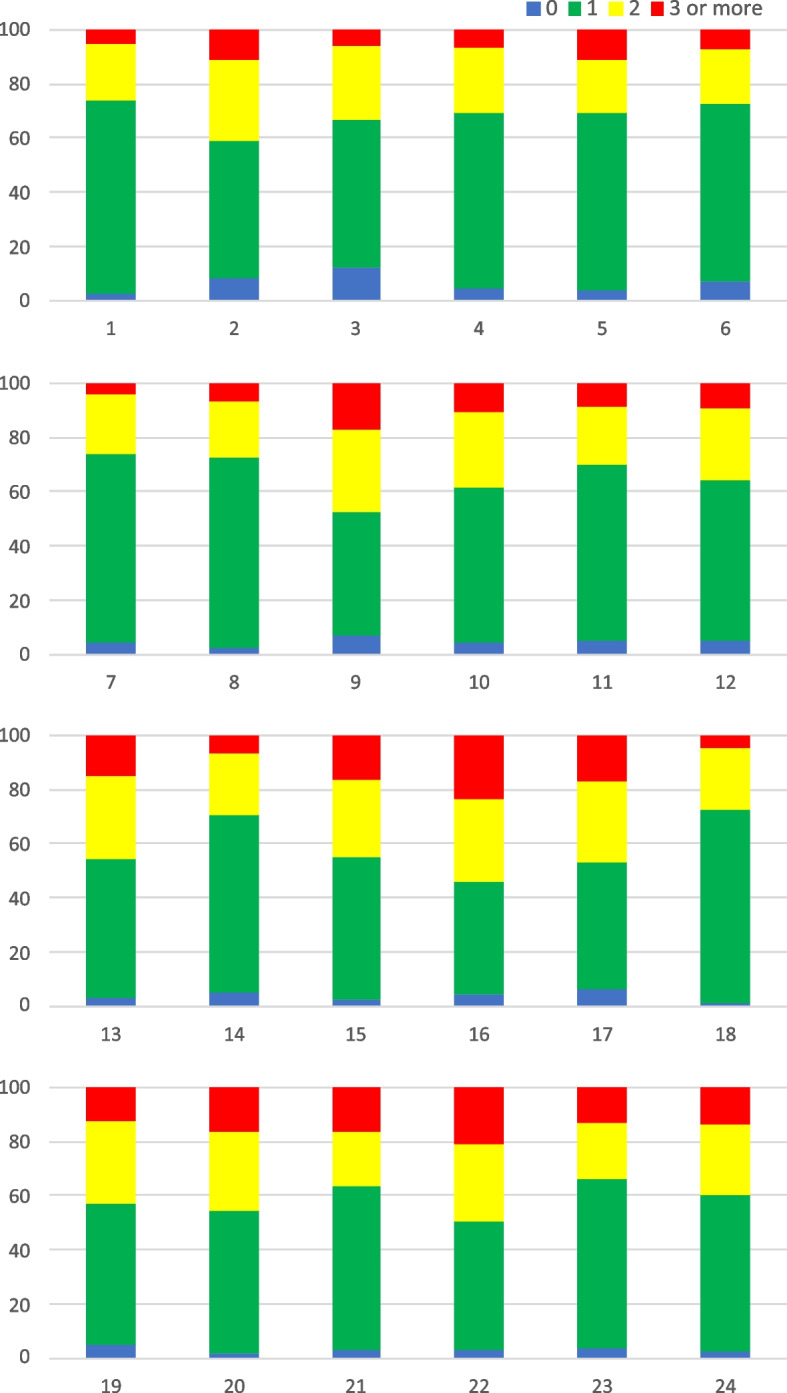
Percentage of the number of times the cards in task F4 were turned over

The Memory Game, task F4, assessed cognitive flexibility and working memory. The number of correct and incorrect pairs for each child was used to validate the scale. Figure 8 demonstrates the percentage for the number of times each card was turned over according to its position in the organization of the cards. It was possible to observe the similarity in the number of times each card was flipped, with a slight preference for the cards that were in the positions closest to the children, that is, the last two rows (from top to bottom).

The structural equation model in this study provides evidence supporting the internal structure and the convergent validity of TDI-FE. However, it does not yield a scoring scheme that is feasible and accessible to less statistically educated professionals that work with children. For this reason, we also developed a simplified formula to compute a general EF score using the results from the F1, F2, F3, and F4 tasks of the TDI-FE. It considers the number of correct answers in tasks F1, F2, and F3, the number of correct pairs in task F4, and the child’s age, according to the formula:

$$EF\;Score=F1\;\left(correct\;answers\right)+\;F2\;\left(correct\; answers\right)+\;F3\;\left(correct\;answers\right)+\;F4\;\left(correct\;pairs\;\right)-Age(years)$$

**Fig. 9:**
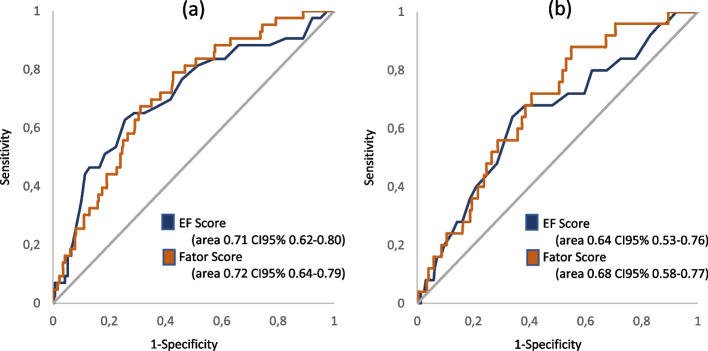
ROC curve of the EF and Factor scores, considering the **a** Cancellation Attention Test and **b** Trail Making Test – Part B as the gold standard

This EF Score yielded a strong and positive correlation (*r* = 0.762; *p* < 0.001) with the factor score obtained from the Structural Equation Model. We then tested the capacity of both the EF Score and the factor score (derived from the Structural Equation Model) to discriminate between children classified as *very low*/*low* versus the remaining children, according to the gold standards CAT (0.71) and TMT - Part B (0.64) scales. The ROC curves displayed in Fig. 9 exhibit a similar performance between the proposed EF Score and the model-produced factor score. In both scales, an EF cut-off score of 37 points was associated with approximately 70% sensitivity and 60% specificity. This supports the diagnostic accuracy of the newly proposed battery.

## Discussion

Executive Functions begin to develop in early childhood and are essential for the development of other skills later in life [3, 11]. Thus, it is important to assess these skills in children in both clinical and school settings to identify potential difficulties early on. The development of instruments with solid psychometric properties for the Brazilian population is crucial and depends on appropriate statistical methods. Thus, psychometrics can collaborate with neuropsychology to identify evidence and test theoretical models of EF [37]. Most instruments of EF assessments in children are adaptations of indicators that were initially developed for adults. Consequently, the assessment may be more difficult and less accurate, as the greater the difference between the assessed individual and the culture (in which the measure was developed), the greater the likelihood that the test score will not represent the construct it proposes to measure [21, 38]. In Brazil, given the lack of accessible and easy to apply assessment instruments for children in early childhood education, TDI-FE was developed as part of a test battery composed of language and motor function assessment instruments. This test battery will be made available to Brazilian researchers free of cost.

This study aimed to test the proposed one-factor structure of the TDI-FE, control for a fatigue factor, estimate relationships with external variables, and test its diagnostic accuracy. Initially, CFA was applied to test the hypothesis that a single factor of executive functions would explain the performance across the set of TDI-FE tasks, the model did not achieve a good fit to the data. On the other hand, factor loadings of each indicator were positively and theoretically coherent, suggesting the tasks indeed measure a common executive factor. As discussed further, the lack of fit was due to the presence of a non-modeled fatigue factor. Even if there is growing consensus about the existence of several basic components of EFs, especially in adults [2, 6, 8, 18, 38, 39], studies suggest as well that a more general component impacts cognitive functioning among children, thereby justifying the single-factor model in this study [38].

We hypothesized that, despite capturing a general executive functions factor, the last TDI-FE tasks might have performance also influenced by a fatigue factor. As reported elsewhere, there is a risk that assessment tests that are longer could generate attrition and demotivation among younger participants [3, 21]. Accordingly, the residuals and modification indices indicated non-negligible residual correlations between the later tasks applied to the children. By including a fatigue factor, we found a better fit for the hypothesized one-factor model of the TDI-FE. With this last model, we detected a compensatory effect across tasks, in which factor loadings for the executive functions decreased, while factor loadings on the fatigue factor increased, especially for the last two items of task F3. These findings are important because they confirm that performance of younger individuals may decrease as a function of fatigue when taking longer tests as in a battery. This might be true to many other instruments other than the TDI-FE. Professionals and practitioners should be careful in making recommendations based on the performance of children in the last of a series of tasks applied to measure executive functions. The adequacy of the test corroborates one of the elements of neuropsychological assessment currently in discussion: it is difficult to measure a task without the influence of the environment involved in the task [2, 38]. The test was applied in a school environment and during school hours, which may have influenced the Fatigue factor.

We also tested the relationships of the EF factor with external variables and compared the coefficients against those of the fatigue factor with the same factors. The structural equation model revealed that only the EF factor was connected to scores on the largely validated scales CAT [31], and TMT/TMTP [32, 33]. These findings support the convergent validity of the newly proposed TDI-FE as an informative battery of executive functioning among children. Moreover, we should stress that both EF and fatigue factors were positively predicted by age. While this pattern is consistent with the established developmental pattern that older children have improved executive functioning, it also indicates that these children are more likely to lose motivation when taking cognitive tests. Therefore, we recommend that testing sessions with older children be planned to contain fewer tests or that longer batteries be applied during more than one testing session.

Finally, our ROC curve analyses provide support for the diagnostic accuracy of the TDI-FE battery. The results indicated that TDI-FE is a fairly sensitive instrument that can identify potential difficulties in the development of EFs. To make the scale easier to apply for health and education professionals, a score with a cut-off point in TDI-FE was created to enable more appropriate screening and referrals for diagnostic evaluation.

Although the instrument was developed with a layout designed for children, the digital format would be sometimes more appealing, especially nowadays [2]. This may be considered a limitation of the study. Since there is a lack of access to electronic devices in some areas of Brazil [21]. To make the instrument easily accessible to health and education professionals, and to facilitate its application in clinical, research, and school settings, the physical form of the model was chosen. As technology becomes more accessible, the availability of neuropsychological assessments via computers and tablets may expand the application of these assessments in Brazil, following the international trend [38]. Another limitation considered is that our study did not measure the three EF components separately. However, EFs involve the use of different skills concomitantly and we have developed a quick-to-use screening instrument trying to assess several skills in a single item.

## Conclusions

The current study is original in the application of complex structural equation modeling techniques to data collected using EF tasks. As discussed elsewhere, typical psychometric investigations of neuropsychological instruments focus more on content and external relationships than on internal structure [37]. The study also proved the need to control for response biases such as fatigue in the latent variable models of EF. The new TDI-FE demonstrated sound psychometric properties and diagnostic accuracy, then consisting of an efficient alternative for the assessment of EFs in early childhood education. The TDI-FE is notable because of its low cost and easy application, and it might fulfill a need for instruments for individuals from different contexts at this stage of development in Brazil [21]. Further research with private school students and clinical groups can be conducted to contribute to the findings of this study.

The early detection of difficulties in EF development is fundamental because it is a major predictor of the child’s success throughout the school years, and it is more critical for school readiness than math, early reading, and intelligence quotient. Thus, this study aimed to meet an existing demand by proposing the development of a sensitive, standardized, and accessible screening device for Brazilian children which can be adapted to other countries and languages.

## Data Availability

The datasets generated during and/or analyzed during the current study are available from the corresponding author on reasonable request.

## Abbreviations

CAT: Cancellation Attention Test
CFA: Confirmatory Factor Analysis
CFI: comparative fit index
CTT: Classical Test Theory
EFs: Executive Functions
ICF: Informed Consent Forms
IRT: Item response theory
RMSEA: Root mean square error of approximation
TDI: Bateria Multidisciplinar de Triagem do Desenvolvimento Infantil
TDI-FE: Executive Function Assessment Instrument
TLI: Tucker–Lewis Index
TMT: Trail Making Test
TMTP: Trail Making Test for Preschoolers
WLSMV: Weighted least squares means and variance adjusted
